# Antibiotic resistance, virulence determinants and production of biogenic amines among enterococci from ovine, feline, canine, porcine and human milk

**DOI:** 10.1186/1471-2180-13-288

**Published:** 2013-12-10

**Authors:** Esther Jiménez, Victor Ladero, Irene Chico, Antonio Maldonado-Barragán, María López, Virginia Martín, Leonides Fernández, María Fernández, Miguel A Álvarez, Carmen Torres, Juan M Rodríguez

**Affiliations:** 1 Departamento de Nutrición, Bromatología y Tecnología de los Alimentos, Universidad Complutense de Madrid, Ciudad Universitaria, Avda. Puerta de Hierro, Madrid s/n. 28040, Spain; 2 Instituto de Productos Lácteos de Asturias (IPLA-CSIC), Paseo Río Linares s/n 33300, Villaviciosa, Spain; 3 Departamento de Biotecnología de Alimentos, Instituto de la Grasa-CSIC, Sevilla 41012, Spain; 4 Área de Bioquímica y Biología Molecular, Universidad de La Rioja, Logroño, Spain; 5 Probisearch. c/Santiago Grisolía, Tres Cantos 2. 28760, Spain

**Keywords:** *Enterococcus*, Milk, Mammals, Virulence, Antibiotic resistance, Biogenic amines

## Abstract

**Background:**

Recent studies have shown that mammalian milk represents a continuous supply of commensal bacteria, including enterococci. The objectives of this study were to evaluate the presence of enterococci in milk of different species and to screen them for several genetic and phenotypic traits of clinical significance among enterococci.

**Results:**

Samples were obtained from, at least, nine porcine, canine, ovine, feline and human healthy hosts. Enterococci could be isolated, at a concentration of 1.00 × 10^2^ -1.16 × 10^3^ CFU/ml, from all the porcine samples and, also from 85, 50, 25 and 25% of the human, canine, feline and ovine ones, respectively. They were identified as *Enterococcus faecalis*, *Enterococcus faecium*, *Enterococcus hirae*, *Enterococcus casseliflavus* and *Enterococcus durans*. Among the 120 initial enterococcal isolates, 36 were selected on the basis of their different PFGE profiles and further characterized. MLST analysis revealed a wide diversity of STs among the *E. faecalis* and *E. faecium* strains, including some frequently associated to hospital infections and novel STs. All the *E. faecalis* strains possessed some of the potential virulence determinants (*cad, ccf*, *cob*, *cpd*, *efaA*_*fs*_, *agg2*, *gelE*, *cylA*, *esp*_*fs*_) assayed while the *E. faecium* ones only harboured the *efaA*_*fm*_ gene. All the tested strains were susceptible to tigecycline, linezolid and vancomycin, and produced tyramine. Their susceptibility to the rest of the antimicrobials and their ability to produce other biogenic amines varied depending on the strain. Enterococci strains isolated from porcine samples showed the widest spectrum of antibiotic resistance.

**Conclusions:**

Enterococci isolated from milk of different mammals showed a great genetic diversity. The wide distribution of virulence genes and/or antibiotic resistance among the *E. faecalis* and *E. faecium* isolates indicates that they can constitute a reservoir of such traits and a risk to animal and human health.

## Background

Natural lactation provides a wide variety of short- and long-term health benefits, being a critical period for mammals’ growth and development; in fact, precocious weaning is associated with high mortality and morbidity rates, particularly in those species in which IgG transfer mainly occurs through maternal milk [1]. Fresh mammalian milk from a given species usually fulfils the nutritional requirements of the neonates of such species and, also, protects them against infectious diseases.

This protective effect is due to the combined action of a variety of protective factors present in colostrum and milk, such as immunoglobulins, immunocompetent cells, fatty acids, polyamines, oligosaccharides and peptides [2-5]. In addition, it has been recently shown that these biological fluids are the vehicle for a variety of commensal, mutualistic or potentially probiotic bacteria [6-11].

The mammalian milk microbiota seems dominated by staphylococci and streptococci [12-14] but it also contains lactic acid bacteria, including enterococci [7,12,15,16]. Enterococci become normal components of the mammalian gastro-intestinal tract soon after birth [17,18]. Some strains have even been proposed for the production of fermented foods or used as human and animal probiotics. However, enterococci are opportunistic pathogens that may cause a range of different infections in animals and humans, including urinary tract infections, mastitis, sepsis, and endocarditis, particularly in hosts with underlying diseases and in neonates [19-21]. Virulence, antibiotic resistance or gene transfer potential have been considered as strain-specific properties within enterococci [22,23]. Recently, genome sequencing of a high number of diverse *Enterococcus faecium* strains has been applied to resolve the lineage responsible for epidemic and/or multidrug-resistant infections from other strains, and to measure the evolutionary distances between groups [24]. Such approach has shown that each evolutionary bifurcation has been accompanied by the acquisition of new metabolic and colonization traits on mobile elements and genome remodeling associated with the insertion and movement of such elements. As a result, diversity within such enterococcal species, in terms of sequence divergence as well as gene content, may span a range usually associated with speciation [24].

The use of antimicrobial agents in the modern farm industry has created a reservoir of resistant enterococci in food animals and in food of animal origin [25,26]; these enterococci are likely to contribute resistance and virulence-associated genes to enterococci inhabiting pets and human hosts since such genes appear to spread freely between enterococci from different reservoirs, irrespective of their apparent host association [27,28]. Moreover, enterococci are one of the groups of bacteria mainly responsible for the accumulation of biogenic amines (BAs) -especially tyramine and putrescine- in fermented dairy foods. BAs are nitrogenous compounds formed by amino acid decarboxilation, with important physiological functions in mammals, as brain activity, immune response, cell growth and differentiation, etc. However, the consumption of food contaminated with BAs provokes several toxic effects, particularly in people who have impaired the detoxification system [29].

Since milk constitute one of the first sources of enterococci to the mammalian gut, the objectives of this study were, first, to evaluate the presence of enterococci in milk of healthy hosts belonging to different mammals’ species, including food animal species (sow, ewe), pets (bitches, queens) and women, and, subsequently, to screen them for several genetic and phenotypic traits of clinical significance among enterococci.

## Methods

### Source and isolation of bacterial isolates

Milk samples were obtained from porcine (intensive farming), canine, ovine (extensive farming), feline and human hosts (Table 1) living in the same geographical area and that fulfilled the following criteria: (a) healthy individuals without present or past underlying conditions; (b) normal pregnancy; and (c) absence of perinatal problems in the mother and in the infant/offspring. For each species, a total of 8 samples (from different individuals) were collected, with the exception of porcine milk (9 samples). The protocol for milk collection from the animals was approved by the Ethical Committee on Animal Experimentation of Universidad Complutense de Madrid (Spain) and, in addition, all the animals’ owners gave their consent to sampling and analysis. All human volunteers gave written informed consent to sample collection and analysis, which were approved by the Ethical Committee of Hospital Clínico of Madrid (Spain).

**Table 1:** Enterococcal concentration (CFU/ml) in milk samples of different mammalian and strains isolated from each sample

**Species**	**Sample**	**Concentration**	***E. faecalis***	***E. faecium***	***E. durans***	***E. hirae***	***E. casseliflavus***
Porcine	P1	8.00 × 10^2^	ECA3	ECA2B	-	-	-
	P2	9.02 × 10^2^	ECB1	ECB4	-	-	-
	P3	1.16 × 10^3^	ECC5	ECC2A	-	ECC1	-
	P4	1.04 × 10^3^	ECD1^a^	ECD3	-	-	-
			ECD2				
	P5	8.38 × 10^2^	ECE1^a^	-	-	-	-
	P6	8.72 × 10^2^	-	ECF2	-	-	-
				ECF5			
	P7	9.46 × 10^2^	ECG2^b^	-	-	ECG1	-
	P8	8.68 × 10^2^	ECH1^c^	-	-	-	-
			ECH6				
	P9	8.28 × 10^2^	ECI1^b^	-	-	-	-
			ECI3^c^				
Canine	C1	3.02 × 10^2^	PKG12	-	-	-	-
	C2	2.58 × 10^2^	PRA5	-	-	-	-
	C3	2.62 × 10^3^	-	PGAH11	-	-	-
	C4	1.24 × 10^2^	-	PKB4	-	-	-
Ovine	O1	7.22 × 10^2^	EOA1	-	-	EOA2	-
	O2	8.00 × 10^2^	EOB6A	-	-	-	EOB3
							EOB5
Feline	F1	6.20 × 10^2^	-	-	-	EH11	-
	F2	5.14 × 10^2^	G8-1 K	-	-	-	-
Human	H1	1.00 × 10^2^	-	-	C2341	-	-
	H2	1.22 × 10^2^	-	-	C1943	-	-
	H3	2.12 × 10^2^	C1252	-	-	-	-
	H4	1.66 × 10^2^	C901	-	-	-	-
	H5	1.54 × 10^2^	-	C656	-	-	-
	H6	2.32 × 10^2^	-	-	C654	-	-
	H7	2.16 × 10^2^	-	-	C502	-	-
TOTAL	29		15^d^	9	4	4	2

^a^Isolates ECD1 and ECE1 are identical; ^b^Isolates ECG2 and ECI1 are identical; ^c^Isolates ECH1 and ECI3 are identical. ^d^Number of different *E. faecalis* strains.

Milk samples (~5 ml from sows, ewes and women; ~3 ml from the remaining species) were collected in sterile tubes by manual expression using sterile gloves. Previously, nipples and surrounding skin were cleaned with soap and sterile water, and soaked in chlorhexidine (Cristalmina, Salvat, Barcelona, Spain). The first drops (~1 ml) were discarded. The milk samples were obtained at day 7 after delivery and kept at 4°C until delivery to the laboratory, which happened within the first three hours after collection.

Samples (the original samples but, also, three serial decimal dilutions of each one in peptone water) were plated (100 μl) in triplicate onto Kanamycin Esculin Azide (KAA, Oxoid, Basingstoke, UK) agar plates. Parallel, and to evaluate potential faecal contamination, the samples were also cultured on Violet Red Bile Agar (VRBA; Difco, Detroit, MI) agar plates; all the plates were aerobically incubated at 37°C for 24 h. In both growth media, the lower limit of detection was 10 CFU (colony-forming units)/ml.

### Identification of bacterial isolates

The potential enterococal isolates (black colonies growing on KAA agar) were observed by optical microscopy to determine their morphology and Gram staining. Additionally, they were tested for catalase, oxidase and coagulase activities. A single colony of each isolate was suspended in 20 μl of deionized sterile water; 5 μl of the suspension were used as a template for species identification by PCR. First, the gene *ddl*, which encode D-alanine:D-alanine ligases, was used as target following the protocol previously described by Dutka-Malen et al. [30]. The pair of primers E1 (5′-ATCAAGTACAGTTAGTCTT-3′)/E2 (5′-ACGATTCAAAGCTAACTG-3′), allowed to identify *E. faecium* strains, while the second pair F1 (5′-GCAAGGCTTCTTAGAGA-3′)/F2 (5′-CATCGTGTAAGCTAACTTC-3′) is specific for *Enterococcus faecalis*. Identification of the rest of isolates was performed by sequencing the 470 pb fragment of the 16S rDNA gene PCR amplified using the primers pbl16 (5′-AGAGTTTGATCCTGGCTCAG-3′) and mbl16 (5′-GGCTGCTGGCACGTAGTTAG-3′) [31]. The PCR conditions were as follows: 96°C for 30 s, 48°C for 30 s and 72°C for 45 s (40 cycles) and a final extension at 72°C for 4 min. The amplicons were purified using the Nucleospin® Extract II kit (Macherey-Nagel, Düren, Germany) and sequenced at the Genomics Unit of the Universidad Complutense de Madrid, Spain. The resulting sequences were used to search sequences deposited in the EMBL database using BLAST algorithm and the identity of the isolates was determined on the basis of the highest scores (>99%).

### Genetic profiling of the enterococcal isolates

Initially, the enterococcal isolates were typed by Random Amplification of Polymorphic DNA (RAPD) in order to avoid duplication of isolates from a same host. RAPD profiles were obtained using primer OPL5 (5′-ACGCAGGCAC-3′), as described by Ruíz-Barba et al. [32]. Later, a representative of each RAPD profile found in each host was submitted to PFGE genotyping [33]; for this purpose, chromosomal DNA was digested with the endonuclease *Sma*I (New England Biolabs, Ipswich, MA) at 37°C for 16 h. Then, electrophoresis was carried out in a CHEF DR-III apparatus (Bio-Rad) for 23 h at 14°C at 6 V/cm with pulses from 5 to 50 s. A standard pattern (Lamda Ladder PFG Marker, New England Biolabs) was included in the gels to compare the digitally normalized PFGE profiles. Computer-assisted analysis was performed with the Phoretix 1D Pro software (Nonlinear USA, Inc., Durham, NC).

### Multilocus sequence typing (MLST)

Molecular typing of *E. faecalis* and *E. faecium* isolates was performed by MLST. Internal fragments of seven housekeeping genes of *E. faecalis* (*gdh*, *gyd*, *pstS*, *gki*, *aroE*, *xpt* and *yiqL*) and *E. faecium* (*atp*A*, ddl*, *gdh, pur*K, *gyd, pst*S*,* and *adk*) were amplified and sequenced. The sequences obtained were analyzed and compared with those included in the website database (http://efaecalis.mlst.net/), and a specific sequence type (ST) and clonal complex (CC) was assigned [34,35].

### Screening for virulence determinants, hemolysis and gelatinase activity

A multiplex PCR method [15] was used to detect the presence of virulence determinants encoding sex pheromones (*ccf*, *cpd*, *cad*, *cob*), adhesins (*efa*_*Afs*_, *efa*_*Afm*_), and products involved in aggregation (*agg2*), biosynthesis of an extracellular metalloendopeptidase (*gelE*), biosynthesis of cytolysin (*cylA*) and immune evasion (*esp*_fs_). The primers couples used to detect all the genes cited above were those proposed by Eaton and Gasson [22]. The presence of the *hyl* gene (encoding a glycosyl hydrolase) and IS16 (potential marker of hospital associated *E. faecium* strains) was also checked by PCR among *E. faecium* strains as described previously [36,37]. Control strains used in PCR experiments were *E. faecalis* strains F4 (*efaA*_*fs*_ + *gelE* + *agg* + *cylMBA* + *esp* + *cpd* + *cob* + *ccf* + *cad*+), P36 (*efaA*_*fs*_ + *gelE* + *agg* + *cylA* + *esp* + *cpd* + *cob* + *ccf* + *cad*+) and P4 (*efaA*_*fs*_ + *gelE* + *agg* + *cylA* + *cpd* + *cob* + *ccf* + *cad*+), *E. faecium* P61 (*efaAfm* + *esp*+) and *E. faecium* C2302 (*hyl*). PCR conditions were as follows: initial denaturation at 94°C for 5 min; 30 cycles of denaturation at 94°C for 1 min, annealing at 51°C for 30 s and elongation at 72°C for 1.5 min, and a final extension at 72°C for 5 min.

Haemolysin activity was evaluated on Columbia Blood Agar (Oxoid) containing 5% defibrinised horse blood. Single colonies were streaked onto plates and incubated at 37°C for 24 h. Zones of clearing around colonies indicated haemolysin production.

Production of gelatinase was determined on tryptic soy agar plates (Oxoid) supplemented with 3% gelatin. Plates streaked with the strains were incubated at 37°C for 24 h, and cooled at 4°C for 4 h. A clear halo around colonies was considered to be positive indication of gelatinase activity.

### Capacity to produce biogenic amines

The presence of the tyrosine decarboxylase gene (*tdcA*), histidine decarboxylase gene (*hdcA*) and agmatine deiminase cluster (*AgdDI*) was checked by specific PCR using the primers pairs P2-for and P1-rev [38], JV16HC and JV17HC [39], and PTC2 and AgdDr [40], respectively. PCR conditions were those described by the respective authors. Total DNA, obtained as described by [32], was used as template. *E. faecalis* V583, which produce putrescine and tyramine, and *Lactobacillus buchneri* B301, which produce histamine, were used as positive controls.

The enterococcal strains were grown for 24 h in M17 broth supplemented with 10 mM tyrosine (M17T), 13 mM of histidine (M17H) or 20 mM agmatine (M17A) for the detection of tyramine, histamine and putrescine production, respectively. The supernatants were filtered through a 0.2 μm pore diameter membrane, derivatyzed and analysed by thin layer chromatography (TLC) following the conditions described by García-Moruno et al. [41].

### Susceptibility to antibiotics

Minimum inhibitory concentrations (MICs) of 12 antimicrobial agents (ampicillin, gentamicin, streptomycin, quinupristin/dalfopristin, kanamycin, erythromycin, clindamycin, oxytetracycline, chloramphenicol, tigecycline, linezolid and vancomycin) were determined by the E-test (AB BIODISK, Solna, Sweden) following the instructions of the manufacturer. The E-test strips contained preformed antimicrobial gradients in the test range from 0.016 to 256 μg/ml for tetracycline, erythromycin, gentamicin, kanamycin, clindamycin, ampicillin, chloramphenicol, tigecycline, linezolid and vancomycin, from 0.064 to 1.024 μg/ml for streptomycin, and from 0.002 to 32 μg/ml for quinupristin-dalfopristin. Results from the different antibiotic susceptibility tests were interpreted according to the cut-off values and clinical breakpoints proposed by the European Committee on Antimicrobial Susceptibility Testing (EUCAST) while the breakpoints of the Clinical and Laboratory Standards Institute (CLSI) [42] were used for those antibiotics not included in EUCAST.

### Screening for *van* genes

PCR reactions for *vanA* and *vanB* genes were performed as described previously [30,43]. Oligonucleotides used as primers for the amplification of the 732 bp fragment of the *vanA* gene were VanA1 (5′-GGGAAAACGACAATTGC-3′) and VanA2 (5′-GTACAATGCGGCCGTTA-3′), while those used for amplification of the 1,145 bp fragment of *vanB* were VanBfor (5′-GTGCTGCGAGATACCACAGA-3′) and VanBrev (5′-CGAACACCATGCAACATTTC′). *E. faecium* BM4147 (resistant to vancomycin, VanA+) and *E. faecalis* V583 (resistant to vancomycin, VanB+) were used as positive controls. PCR assays for the detection of *vanD*, *vanE* and *vanG* genes in the enterococcal isolates was performed as previously described [44-46].

## Results

### Isolation, identification and profiling of the enterococcal isolates

Colonies were obtained from all the porcine and 7 out of 8 human samples when inoculated onto KAA plates. In contrast, colonies could be isolated from 50% of the canine samples and only from 25% of the feline and ovine ones (Table 1). When bacterial growth was detected, the KAA counts ranged from 1.00 × 10^2^ to 1.16 × 10^3^ CFU/ml (Table 1). No colonies were detected on VRBA plates, which confirmed the hygienic collection of the milk samples.

Five isolates showing a coccoid shape and catalase-negative and oxidase-negative reactions were randomly selected from each sample in which colonies were observed. The 120 isolates were identified to the species level as *E. faecalis*, *E. faecium*, *Enterococcus hirae*, *Enterococcus casseliflavus* or *Enterococcus durans* (Table 1). Among them, *E. faecalis* isolates were the most abundant and, in addition, this was the only enterococcal species present in samples from all the mammalians’ species included in this study. *E. faecium* was found in canine, swine and human milk samples but not in the ovine or feline ones. *E. hirae* was present in ovine, swine and feline milk samples. Finally, *E. casseliflavus* and *E. durans* could be isolated only from ovine and human milk samples, respectively. There was a maximum of three different enterococcal species in a same sample (porcine sample no. P3: *E. faecalis*, *E. faecium* and *E. hirae*), while only one enterococcal species was detected in each of the canine, feline and human samples (Table 1).

RAPD and PFGE profiling revealed that, for each enterococcal species, there was a single strain per sample, with the exception of four porcine and one ovine samples (Table 1). PFGE genotyping also revealed that three *E. faecalis* strains were shared by different porcine samples (Table 1). Based on their different PFGE profiles, 36 enterococcal isolates from milk of the 5 mammalian species were selected subsequently, for further characterization.

### MLST analysis of the *E. faecalis* and *E. faecium* strains

MLST analysis of the *E. faecalis* strains revealed the occurrence of 8 different STs, including one novel ST (ST473) from a canine sample (Table 2). The most frequent clones were ST16, which was found among 4 strains (all of them from porcine origin), and ST9, which was detected among 3 strains (one porcine strain and the two ovine ones). Clone ST200 was shared by two porcine strains while clone ST21 was shared by one porcine and the feline strain.

**Table 2:** **MLST typing, presence of virulence determinants and hemolytic and gelatinase activities among the *E. faecalis *** **strains**

**Origin**	***Strain***	**ST**^**a**^	***cad***	***ccf***	***cob***	***cpd***	***efaA***_***fs***_	***esp***_***fs***_	***agg***_***2***_	***gelE***	***cylA***	**Gelatinase**	**Hemolysis**
Porcine	ECA3	ST21	+	+	+	+	+	+	-	+	-	+	-
	ECB1	ST9	+	+	+	+	+	+	+	+	+	+	+
	ECC5	ST16	+	+	+	+	+	+	+	+	+	+	+
	ECD2	ST16	+	+	+	+	+	+	+	-	+	-	+
	ECE1	ST200	+	+	+	+	+	+	+	+	-	+	-
	ECH6	ST16	+	+	+	+	+	+	+	-	+	-	+
	ECI1	ST200	+	+	+	+	+	+	+	+	-	+	-
	ECI3	ST16	+	+	+	+	+	+	+	+	+	+	+
Canine	PKG12	ST239	+	+	+	+	+	-	-	+	-	+	-
	PRA5	ST473	+	+	+	+	+	-	-	+	-	+	-
Ovine	EOA1	ST9	+	+	+	+	+	+	+	+	+	+	+
	EOB6A	ST9	+	+	+	+	+	+	+	+	+	+	+
Feline	G8-1 K	ST21	+	+	+	+	+	-	+	+	-	-	-
Human	C1252	ST8	+	+	+	+	+	+	-	+	-	+	-
	C901	ST30	+	+	+	+	+	+	+	+	-	+	-
Total	15	15	15	15	15	15	15	12	11	13	7	12	7
Percentage			100	100	100	100	100	80	73	87	47	80	47

^a^ST obtained by MLST typing.

MLST analysis was also performed with the 9 *E. faecium* strains recovered from the different origins. Eight different STs were detected among *E. faecium* strains, five of them known (ST5, ST30, ST183, ST272, ST442 and ST654), and two new STs that presented new allelic combinations (ST882 and ST883, of porcine origin). For one of the *E. faecium* strains it was not possible to determine the ST (Table 3).

**Table 3:** **MLST typing of the *E. faecium *** **strains**

		**Allele**							
**Origin**	**Strain**	***atpA***	***ddl***	***gdh***	***purK***	***gyd***	***pstA***	***adk***	**ST**^**a**^
Porcine	ECA2B	5	5	1	9	1	1	1	ST882^b^
	ECB4	5	2	1	9	1	1	5	ST5 (CC5)
	ECC2A	4	5	8	3	1	20	1	ST272 (singleton)
	ECD3	4	5	9	3	1	20	1	ST183
	ECF2	9	4	12	3	1	20	1	ST883^b^
	ECF5	49	4	-	-	-	20	8	NT^c^
Canine	PGAH11	5	1	1	2	6	1	1	ST442
	PKB4	5	3	1	6	2	2	1	ST30 (singleton)
Human	C656	8	8	8	23	1	27	15	ST654

^a^ST obtained by MLST typing.

^b^New ST types.

^c^NT: non-typeable.

### Occurrence of putative virulence genes

None of the potential virulence determinants (*cad, ccf*, *cob*, *cpd*, *efaA*_*fs*_, *efaA*_*fm*_, *agg2*, *gelE*, *cylA*, *esp*_*fs*_) tested in this study could be detected in any of the *E. durans*, *E. hirae* or *E. casseliflavus* strains. The *E. faecium* strains only harboured the *efaA*_*fm*_ gene, while all the *E. faecalis* strains possessed some potential virulence determinants (Table 2). Sex pheromones determinants (*ccf*, *cpd*, *cad*, *cob*) and the adhesin gene *efaA*_*fs*_ were detected in all *E. faecalis* strains, whereas the rest of the genes were variable on the strains. The *cylA* gene was not detected in any of the *E. faecalis* strains isolated from human, canine and feline milk. All *E. faecium* strains were negative for the *hyl* gene and the IS16 element.

There was a good correlation between presence of *gelE* gene and gelatinase activity and, also, between presence of *cylA* gene and hemolytic activity (Table 2).

### Production of biogenic amines

All the tested strains were positive for the *tdc* gene and were able to produce tyramine (Table 4). In contrast, none of them harbored the *hdc* gene and histamine was accordingly not detected in the cultures (Table 4). All the *E. faecalis* strains contained the genes involved in putrescine biosynthesis and produced putrescine in broth cultures, while the results were negative for the two *E. casseliflavus* strains. The ability to produce putrescine was variable in the other enterococcal species (*E. faecium*, *E. durans* and *E. hirae*), having found both producing and non-producing strains (Table 4). There were only two strains -both belonging to *E. hirae*- in which the gene (*agdDI*) was present, but the production of the corresponding biogenic amine (putrescine) was not detected.

**Table 4:** Detection of gene determinants for the biosynthesis of biogenic amines and production among the enterococcal isolates

					**Putrescine**	
**Origin**	**Species**	***Strain***	**Tyramine**^**a**^	**Histamine**^**b**^	**Gene cluster**	**Production**
Porcine	*E. faecalis*	ECA3	+	-	+	+
		ECB1	+	-	+	+
		ECC5	+	-	+	+
		ECD2	+	-	+	+
		ECE1	+	-	+	+
		ECH6	+	-	+	+
		ECI1	+	-	+	+
		ECI3	+	-	+	+
Canine		PKG12	+	-	+	+
		PRA5	+	-	+	+
Ovine		EOA1	+	-	+	+
		EOB6A	+	-	+	+
Feline		G8-1 K	+	-	+	+
Human		C1252	+	-	+	+
		C901	+	-	+	+
Porcine	*E. faecium*	ECA2B	+	-	+	+
		ECB4	+	-	+	+
		ECC2A	+	-	-	-
		ECD3	+	-	-	-
		ECF2	+	-	-	-
		ECF5	+	-	-	-
Canine		PGAH11	+	-	-	-
		PKB4	+	-	-	-
Human		C656	+	-	-	-
Human	*E. durans*	C2341	+	-	+	+
		C1943	+	-	+	+
		C654	+	-	-	-
		C502	+	-	-	-
Porcine	*E. hirae*	ECC1	+	-	-	-
		ECG1	+	-	+	-
Ovine		EOA2	+	-	+	+
Feline		EH11	+	-	+	-
Ovine	*E. casseliflavus*	EOB3	+	-	-	-
		EOB5	+	-	-	-

^a^Detection of the *tdcA* gene and production of tyramine in broth cultures; ^b^detection of the *hdcA* gene and production of histamine in broth cultures.

### Antibiotic susceptibility and screening for *van* genes

All the enterococcal strains showed susceptibility to tigecycline, linezolid and vancomycin, and exhibited high resistance to kanamycin. Their susceptibility to the rest of the antimicrobials included in this study is shown in Table 5. Most *E. faecalis*, *E. faecium* and *E. hirae* strains were resistant to tetracycline and chloramphenicol. All *E. faecalis* strains showed susceptibility to ampicillin whereas an important number of strains showed resistance to the rest of antibiotics tested. The strains identified as *E. faecium* and *E. hirae* did not present high-level resistance to gentamicin but exhibited high resistance rate towards the rest of antibiotics. Globally, *E. casseliflavus* was the species with a highest susceptibility to the antibiotics tested followed by *E. durans*.

**Table 5:** **Resistance (+) or susceptibility (−) of the enterococcal isolates against clinically-relevant antibiotics**^**a**^

			**Antibiotic**^**b**^							
**Origin**	**Species**	**Strain**	**AM**	**GM**	**SM**	**EM**	**CL**	**QD**	**TC**	**CM**
Porcine	*E. faecalis*	ECA3	-	-	+	+	-	+	+	+
		ECB1	-	-	+	-	+	+	+	+
		ECC5	-	+	+	+	-	+	+	+
		ECD2	-	+	+	+	-	+	+	+
		ECE1	-	-	+	+	+	+	+	+
		ECH6	-	+	+	+	-	+	+	+
		ECI1	-	-	+	+	+	+	+	+
		ECI3	-	+	+	+	-	+	+	+
Canine		PKG12	-	-	+	-	-	-	-	+
		PRA5	-	-	+	-	+	+	-	+
Ovine		EOA1	-	-	+	-	+	+	+	+
		EOB6A	-	-	+	-	+	+	+	+
Feline		G8-1 K	-	-	+	-	+	+	-	+
Human		C1252	-	+	+	-	-	+	+	+
		C901	-	+	+	-	-	+	+	+
Porcine	*E. faecium*	ECA2B	+	-	+	+	-	-	+	+
		ECB4	-	-	+	-	+	+	+	+
		ECC2A	+	-	+	+	-	+	+	+
		ECD3	-	-	+	-	+	-	+	+
		ECF2	+	-	+	+	-	+	+	+
		ECF5	-	-	+	+	-	+	+	+
Canine		PGAH11	-	-	+	+	-	-	+	+
		PKB4	-	-	+	-	-	-	+	-
Human		C656	-	-	-	-	-	+	-	+
Human	*E. durans*	C2341	-	-	-	-	-	-	-	-
		C1943	-	-	+	-	-	+	-	+
		C654	-	-	-	-	-	-	-	-
		C502	+	+	-	+	+	-	-	+
Porcine	*E. hirae*	ECC1	+	-	-	-	-	-	+	+
		ECG1	+	-	-	+	-	-	+	+
Ovine		EOA2	+	-	-	+	+	+	+	+
Feline		EH11	-	-	-	-	-	+	+	+
Ovine	*E. casseliflavus*	EOB3	-	-	-	-	-	+	-	+
		EOB5	-	-	-	-	-	-	-	-

^a^All the enterococcal strains showed susceptibility to tigecycline, linezolid and vancomycin, and exhibited high resistance to kanamycin.

^b^AM: ampicillin; GM: gentamicin; SM: streptomycin; EM: erythromycin; CL: clindamycin; QD: quinupristin/dalfopristin; TC: tetracycline; CM: chloramphenicol.

In relation with the milk origin, *Enterococcus* strains isolated from porcine samples showed the widest spectrum of antibiotic resistance and all the *E. faecalis* strains from such origin displayed resistance to, at least, six of the ten antibiotics tested (Table 5).

Finally, *van* genes could not detected in any *Enterococcus* strains studied in this work.

## Discussion

Enterococci are common inhabitants of the gastrointestinal tract of humans and a wide variety of animals. In this study, the presence of enterococci in milk samples obtained from different mammalian species was investigated. Enterococci were isolated from all the porcine milk samples and from 7 out of 8 human samples, while they were less frequent in the canine, ovine and feline samples. All the strains were identified as *E. faecalis*, *E. faecium*, *E. hirae*, *E. casseliflavus* or *E. durans*. The number of different species in each milk sample was low, ranging from 1 to 3. Similarly, the number of strains was also low and, in fact, each of the canine and human samples contained only one enterococcal strain. PFGE profiling revealed that only some of the porcine samples shared a given strain, which indicates that spread is facilitated in intensive farming settings.

Globally, the results showed that milk from different mammalian species may contain enterococci and, therefore, may constitute a natural source of such microorganisms for the infant/offspring. The KAA counts (<1.16 × 10^3^ CFU/mL) were similar to those reported for hygienically-obtained human milk on MRS plates, a medium also suitable for isolation of enterococci [6,7]. As previously reported for lactobacilli in porcine and canine milk [8,9], the enterococcal pattern observed in the milk samples seems to be restricted to a low number of species and strains, and also to have a high degree of individual variability. To our knowledge, this is the first description of enterococci isolated from fresh milk of healthy canine, feline and porcine hosts. Some *E. faecium* and *E. faecalis* strains from colostrum and milk of healthy women have been described previously [14-16,47]. In relation to ewe’s milk, a pilot study showed that enterococci were present in excess of 2 × 10^2^ CFU/ml in 15% of the samples of unpasteurized milk from goats and ewes in England and Wales [48]. Other study focused on the identification of indigenous lactic acid bacteria in four samples of fresh ewe’s raw milk and four samples of derived artisanal cheese from Argentina revealed that 48% and 59%, respectively, of the isolates obtained belonged to the genus *Enterococcus*[49].

The *E. faecalis* strains analyzed in this work possessed some potential virulence determinants, including all the sex pheromone determinants, but the gene encoding cytolysin (*cylA*) could only be detected in 7 strains. The results for the rest of the enterococcal genes were variable depending on the strains. On the other hand, only the *efaA*_*fm*_ gene could be detected among the *E. faecium* isolates. These results are similar to those obtained in previous studies with enterococcal strains isolated from human colostrum and milk [14-16]. The role of adhesin *EfaA*_*fm*_ in virulence has not yet been demonstrated, in contrast to the Esp surface protein. In the absence of other virulence determinants, presence of *efaA*_*fm*_ seems to have no value as a risk indicator since this gene was also found in 100% of starter *E. faecium* strains with a long record of safe use in food [22]. The results also agree with those obtained in other studies focused on foodborne enterococci in the sense that *E. faecalis* strains harbor multiple virulence determinants with a much higher incidence than in other enterococcal species [23].

A great diversity of *E. faecalis* and *E. faecium* clones were detected circulating in the milk environments of different origins including three that have not been described previously. Some of the clones were common in different animal species as it was the case of *E. faecalis*-ST21, which was detected among porcine and feline isolates, or *E. faecalis*-ST9 among porcine and ovine ones. The sequence types found among the human isolates were only observed in milk samples of this origin. It is of interest to remark that two of the STs detected among *E. faecalis* strains of porcine or feline origin are included in clonal complexes (CC16 and CC21) that are frequently detected in human infections in Europe [50]. In addition, it should be highlighted that the hospital-associated lineages of *E. faecalis* (ST21 and ST16) and *E. faecium* (ST5), identified in milk of porcine origin in this study, have also been detected in the pig farm environment in a recent study [51].

Several food and human isolates belonging to different species of the genus *Enterococcus* had been previously described as BA producers [52]. In fact, tyramine production and a variable ability to produce putrescine is a very common finding among enterococci [40]. However, to our knowledge, no histamine-producing enterococci strains have been described so far and have not been found in this work, either. Although it has been generally assumed that the ability to produce BAs is a strain-dependent characteristic, it has been recently described that tyramine biosynthesis is a species-level characteristic in *E. faecalis*, *E. faecium* and *E. durans*[40]. The same work suggests that putrescine biosynthesis by the agmatine deiminase pathway is also a species-level characteristic in *E. faecalis*. Since all the strains tested in this study showed ability to synthesize tyramine, and all the *E. faecalis* strains produced putrescine (Table 4), the results obtained are consistent with the fact that they are species-level characteristics. Moreover, all *E. hirae* and *E. casseliflavus* strains were also tyramine producers. Although further work is required, tyramine-production could also be a species-level characteristic of these species. In any case, the ability to produce tyramine is widespread in the genus *Enterococcus*. With respect to putrescine, the results are more variable. While all the *E. faecalis* were putrescine producers, only some *E. faecium* and *E. hirae* strains and none *E. casseliflavus* produced it. Genomic studies on *E. faecium* suggest that such ability could have been acquired through horizontal gene transfer [40].

The presence of BA-producing enterococci in human milk evidences the need to research if they can produce BAs in the milk, or subsequently in the gastrointestinal tract, and therefore be considered a health risk. In fact, it has been shown that tyramine-producing *E. durans* strain isolated from cheese is able to produce tyramine under conditions simulating transit through the gastrointestinal tract [53]. The milk used for the production of fermented dairy products (cows, ewes and goats) deserves also further research, since the presence of BA-producing enterococci may be responsible for the accumulation of toxic BAs concentrations in foods [54].

The E-test was used to determine the resistance pattern of the enterococcal strains against 10 clinically-relevant antimicrobials. The antibiotic resistance spectrum was wider among the *E. hirae*, *E. faecium* and, particularly, *E. faecalis* strains. In relation to the source of the samples, those isolated from porcine milk seemed to be of particular concern. Antibiotic resistance is an important factor for the safety evaluation of enterococci because it can be acquired and/or transferred to other bacteria by gene transfer. The major differences in the rate of resistant enterococci in porcine herds among different countries are most probably due to differences in the usage of antimicrobial agents [55].

Vancomycin-resistant enterococci (VRE) initially emerged as a relevant Public Health threat due to the use in the past of the glycopeptide avoparcin as growth promoter in animal feed. Once avoparcin was banned, the persistence of VRE was associated to co-selection of *van* genes and genes conferring resistance to other antibiotics (such as erythromycin) due to the intensive use of other antibiotics, such as tylosin [56]. After the ban of antibiotics as growth promoters in all European Union countries (July 1999), Aarestrup [57] speculated that occurrence of VRE among pigs would decrease in the following years. In this study, none of the strains was resistant to vancomycin, an antibiotic commonly used for infections caused by multidrug-resistant bacteria, although most of the *E. faecalis* strains isolated from porcine milk were resistant to erythromycin.

All our *E. faecalis*, *E. faecium* and *E. hirae* strains of food animals (porcine and ovine) were resistant to tetracycline, which has been widely used for therapy in food animals in many countries, including Spain; this usage also could have contributed to the successful persistence of *tet* genes. A comparison between antibiotic resistance among enterococci isolated from pigs in Sweden, Denmark and Spain showed that *tet* (L) and *tet* (S) genes were more frequently found among isolates from Spain [55].

Globally, frequent occurrences of antibiotic-resistant enterococci have been observed among food animals, and it has been suggested that these animals may be a reservoir of resistant enterococci and resistance genes capable of transferring to humans through the food chain [58]. Antimicrobial resistance genes appear to spread freely between enterococci from different reservoirs, irrespective of their apparent host association [58].

Therefore, continuous surveillance of antimicrobial resistance in enterococci from humans, animals and foods of animal origin is essential to detect emerging resistance and new infections [26]. As an example, an outbreak of infective mastitis due to *E. faecalis* was recently reported in an intensive sheep farm in Italy. Forty-five out of the 48 *E. faecalis* isolates showed the same multi-drug resistance pattern and had a clonal origin. This was the first reported case of ewe’s mastitis caused by *E. faecalis*[59]. Such strains could arrive to the human food chain through the consumption of cheeses elaborated with raw ewe’s milk.

Pets can also be a source of enterococci and enterococcal resistance genes to humans and other animals and vice versa. Recent results suggest that direct and frequent contact with dogs may significantly shape the composition of our microbial communities [60]. The widespread occurrence of ampicillin-resistant clones in dogs is worrying since these animals may spread such clones among humans due to the close relationships that are usually established between dogs and humans [61,62]. Due to this risk of zoonotic transfer, it has been suggested that pets used to promote the recovery of patients (pet therapy) may pose a risk to such patients if the dogs are not previously screened for the presence of such enterococcal clones [61]. Similarly, it has been reported that dogs leaving the veterinary intensive care unit (ICU) carry a very large multi-drug resistant enterococcal population with capacity for horizontal gene transfer [63]. As a consequence, the authors recommended restriction of close physical contact between pets released from ICUs and their owners to avoid potential health risks [63].

## Conclusions

Milk from different mammalian species may contain enterococci. The wide distribution of virulence genes and/or antibiotic resistance among *E. faecalis* and *E. faecium* strains isolated from such source indicates that they can constitute a reservoir of such traits for the infant/offspring gut and, as a consequence, a potential risk to animal and human health. In fact, some STs detected among *E. faecalis* strains isolated from porcine or feline samples in this study belong to clonal complexes (CC16 and CC21) frequently associated to hospital infections in Europe.

## Competing interests

The authors declare that they have no competing interests.

## Authors’ contributions

EJ, IC, AMB, VM and, LF isolated, identified and characterized the strains. VL and MF performed the BA analysis. ML and CT carried the MLST analysis. CT, MAA and JMR designed experimental procedures. EJ, JMR, MAA and CT drafted the manuscript. All authors read, revised and approved the manuscript.
